# A comparative study of cyanoacrylate-based tissue adhesive and surgical sutures on marginal flap stability following coronally advanced flap

**DOI:** 10.1007/s00784-023-05390-8

**Published:** 2023-12-20

**Authors:** Andreas Pabst, Philipp Becker, Robert Kuchen, Sven Schumann, Adrian Kasaj

**Affiliations:** 1 Department of Oral and Maxillofacial Surgery, Federal Armed Forces Hospital, Rübenacher Str 170, 56072 Koblenz, Germany; 2 Department of Oral and Maxillofacial Surgery, University Medical Center Mainz, Augustusplatz 2, 55131 Mainz, Germany; 3 Institute for Medical Biostatistics, Epidemiology and Informatics (IMBEI), University Medical Center Mainz, Obere Zahlbacher Str. 69, 55131 Mainz, Germany; 4 Institute of Anatomy, University Medical Center Mainz, Johann-Joachim-Becher-Weg 13, 55128 Mainz, Germany; 5 Department of Periodontology and Operative Dentistry, University Medical Center Mainz, Augustusplatz 2, 55131 Mainz, Germany

**Keywords:** Cyanoacrylate, Tissue adhesive, Periodontal surgery, Coronally advanced flap, Tensile force

## Abstract

**Introduction:**

The present study evaluated the biomechanical characteristics of cyanoacrylate-based tissue adhesive (TA) compared to surgical sutures in coronally advanced flap (CAF) procedures using an ex-vivo model.

**Material and methods:**

Thirty-six half-pig mandibles were divided into three groups, *n*=12 each: (I) CAF fixed with sutures (sling and tag suture technique), (II) CAF fixed with TA, and (III) CAF fixed with sutures and TA. At mandibular premolars, gingival recession defects extending 3 mm apical to the cemento-enamel junction (CEJ) were created. CAF procedures were performed using a split-full-split approach, with coronal advancement of the flap to 1 mm above the marked CEJ and stabilization according to the respective groups I–III. Marginal flap stability against pull-of forces (maximum tensile force) was measured with a universal material testing machine until the CEJ became visible.

**Results:**

The comparison between groups I–III demonstrated a significantly increased maximum tensile force for the TA (II) compared to the suture group (I) (*p*<0.001). A significantly increased maximum tensile force was found for the suture and TA (III) compared to the suture group (I) (*p*<0.001). There was also a significantly increased maximum tensile force in the suture and TA (III) compared to the TA group (II) (*p*<0.001).

**Conclusion:**

The results suggest that cyanoacrylate-based TA can increase marginal flap stability compared to sutures in CAF procedures.

**Clinical relevance:**

Cyanoacrylate-based TA can be considered a useful and valuable adjunct to conventional suturing techniques in periodontal plastic surgery, especially in cases where high flap stability is required. The results of this ex-vivo study can only be transferred to the clinical situation with limitations. Clinical long-term follow-up data must be generated.

## Introduction

Gingival recessions are defined as a displacement of the gingival margin apical to the cemento-enamel junction (CEJ) and are accompanied by clinical attachment loss [1]. Aside from esthetic limitations, gingival recessions are associated with dentine hypersensitivity, root caries, non-carious cervical lesions, and compromised biofilm control. The cause is usually multifactorial, with traumatic tooth-brushing techniques and periodontal inflammation being the main reasons for the development of gingival recession. In addition, local anatomical factors, such as a lack of keratinized tissue height (KT) and thin gingival tissue, high frenulum insertion, but also smoking and general diseases (e.g., diabetes mellitus) or prosthetic factors may contribute to their emergence [2, 3]. Gingival recessions can be classified according to gingival and tooth-related factors. These include recession depth, KT, gingival thickness (GT), interproximal and buccal attachment loss, and the appearance of the exposed root surface [1]. The current classification according to Cairo et al. distinguishes three types of recession defects. Recession type 1 (RT 1) refers to recessions that exhibit no loss of interproximal attachment. Interproximal attachment loss occurs in recession type 2 (RT 2). However, the amount of interproximal attachment loss is less than or equal to the buccal attachment loss. The amount of interproximal attachment loss in recession type 3 (RT 3) is higher than the buccal attachment loss [1]. This classification can be further refined by considering the condition of the exposed root surface according to Pini-Prato et al. Hereby, the presence (A) or absence (B) of an identifiable CEJ and presence (+) or absence (−) of dental surface discrepancy (step > 0.5 mm) are assessed, resulting in four different classes (A+, A−, B+, B−). In this classification system, restorative aspects are the focus due to different dental surface defects. A detectable CEJ is a prerequisite for diagnosis and correct positioning of a coronally advanced flap (CAF). A dental surface step can further complicate flap and graft positioning and reduce postoperative recession coverage [4]. Thus, a gingival recession should ideally be described by both classification systems (e.g., RT 1, B−), plus information on periodontal phenotype and recession depth.

The first step in treating gingival recession is always the identification and, if possible, the elimination of etiologic factors related to recession defects. To cover gingival recessions, a surgical procedure that has been well established for decades is the CAF [5]. In the classic CAF procedure, a vertical releasing incision is performed mesial and distal of the affected tooth. The flap is elevated split-thickness so that the alveolar bone remains covered with soft tissue [2] . After mechanical treatment of the root surface, the prepared flap is moved coronally to cover the gingival recession and fixed with sutures. The technique was developed in 1926 by Norberg as an esthetic surgical procedure to cover gingival recessions and was repeatedly advanced and modified [6–10]. Nowadays, the modified CAF technique using a “split-full-split” approach (de Sanctis and Zucchelli) is widely used [7]. The success of the CAF depends on general, patient-specific, local, but also on surgical factors. Thus, the technique-related factors, such as flap thickness, flap tension, the final position of the gingival margin, and the wound closure technique in general are of great importance for the final outcomes of a CAF and other oral wound closure procedures [11, 12]. Indeed, proper closure and adaptation of the flap with appropriate suturing appear to be a prerequisite for achieving optimal surgical outcomes [13]. For this purpose, various procedures and suturing techniques have been proposed to stabilize a CAF, such as the simple interrupted suture, sling suture, and sling and tag suture [6, 14–16]. Suspended sutures that are passed over interproximal composite points or orthodontic buttons can also be helpful for improved flap stabilization [17, 18]. Regarding the suturing technique, it has been demonstrated that a single sling and sling and tag (SAT) suture technique is superior to a conventional simple interrupted suture in terms of flap stability [6]. In addition, the type of suture material should be considered depending on the thickness of the tissue and the surgical procedure itself [19]. As an alternative to conventional sutures, cyanoacrylate-based tissue adhesives have been proposed as a suitable alternative for surgical wound closure and can be used for different indications. These include free gingival graft stabilization, wound closure at the palatal donor site, fixation of bone grafts, fixation of periodontal flaps, closure of Schneiderian membrane perforations, and intraoral wound closure [20–28]. Modern tissue adhesives are based on a combination of n-butyl and 2-octyl cyanoacrylates. Butyl cyanoacrylate (e.g., Histoacryl®) can reduce wound closure time, while octyl cyanoacrylate (e.g., Dermabond®) can lead to a higher strength of the wound closure [29, 30]. Chemically, cyanoacrylate tissue adhesive (CTA) is initially in the form of monomers, polymerizes in a humid environment by an exothermic reaction upon contact with anionic compounds, and adheres to tissue after curing. Hemostatic and bacteriostatic effects should be emphasized as advantages of CTA, which can lead to improved bleeding control and less postoperative wound infection [31, 32]. In addition, the application of CTA is less traumatic and less painful. In the case of non-infected wounds, a saliva-proof wound closure could help to prevent surgical site infection. Compared to conventional sutures, better results of CTA in terms of infection, hemostasis, time, and pain were reported [33–36]. A recent study demonstrated that CTA is a suitable alternative to conventional sutures for wound closure in various mucoperiosteal flaps and around dental implants from a biomechanical aspect [12]. The aim of this study was to evaluate the effectiveness of a cyanoacrylate-based tissue adhesive in comparison to conventional sutures for CAF stabilization in root coverage procedures.

## Material and methods

### Tissue adhesive

A commercially available tissue adhesive (PeriAcryl® 90 HV; GluStitch Inc., Delta, Canada) was used for this study. According to the manufacturer’s information, PeriAcryl® 90 HV is a high-viscosity tissue adhesive chemically based on n-butyl- and 2-octyl-cyanoacrylate and was originally developed for intraoral application. The n-butyl-cyanoacrylate component is responsible for the adhesive’s fast-setting characteristics and the 2-octyl-cyanoacrylate component for the flexible dressing^1^. Periacryl® 90 HV was strictly used according to the manufacturer’s protocol and information. Briefly, tissue adhesive was stored at room temperature (RT). Shortly before use, tissue adhesive was filled into an autoclavable tray (GluStitch Inc.) and loaded into small pipettes (GluStitch Inc.) designed for intraoral application. After the application of an initial viscous layer on oral soft tissues, a soaked cotton gauze was used to accelerate the polymerization process. A total of three layers were applied until a compact and uniform stable structure was achieved. The interval between each application was at least 30 s [37].

### Ex vivo pig mandible model

In total, 36 half-pig mandibles from domestic pigs were ex-vivo used for the study. Mandibles originated from pigs of the very same age and size. The age of the pigs ranged between 6 and 8 months, and the weight ranged between 200 and 220 pounds. There was no differentiation concerning the mandibles originating from male and female animals. Pig mandibles were received from a local butcher specialist trade. All mandibles were approximately the same magnitude. Before the experiments, the number and the integrity of all teeth of the permanent dentition were controlled at each mandible. Further, the integrity of the oral soft tissues including the keratinized attached tissue and alveolar mucosa was checked. In addition, there was a control for preexisting gingival recessions. In case of any tooth or soft tissue damage, the particular mandible was excluded from the experiments and replaced by another one. Immediately before surgery, mandibles were washed with water at RT and carefully dried with fine cellulose papers to remove blood residues and the smear layer from the teeth and the oral tissues. Finally, mandibles were randomly divided into three experimental groups, 12 mandibles each: (I) coronally advanced flap (CAF) fixed with sutures, (II) CAF fixed with tissue adhesive, and (III) CAF fixed with sutures combined with tissue adhesive.

### Coronally advanced flap model

**Fig. 1:**
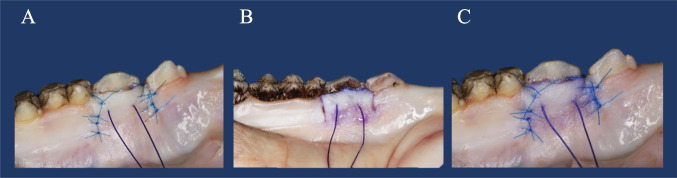
**A** CAF fixed with sling and tag suture. Vertical releasing incisions were closed with three single interrupted sutures, respectively. **B** CAF fixed with three layers of tissue adhesive. **C** CAF fixed with the combination of both procedures, sling and tag suture and tissue adhesive

**Fig. 2:**
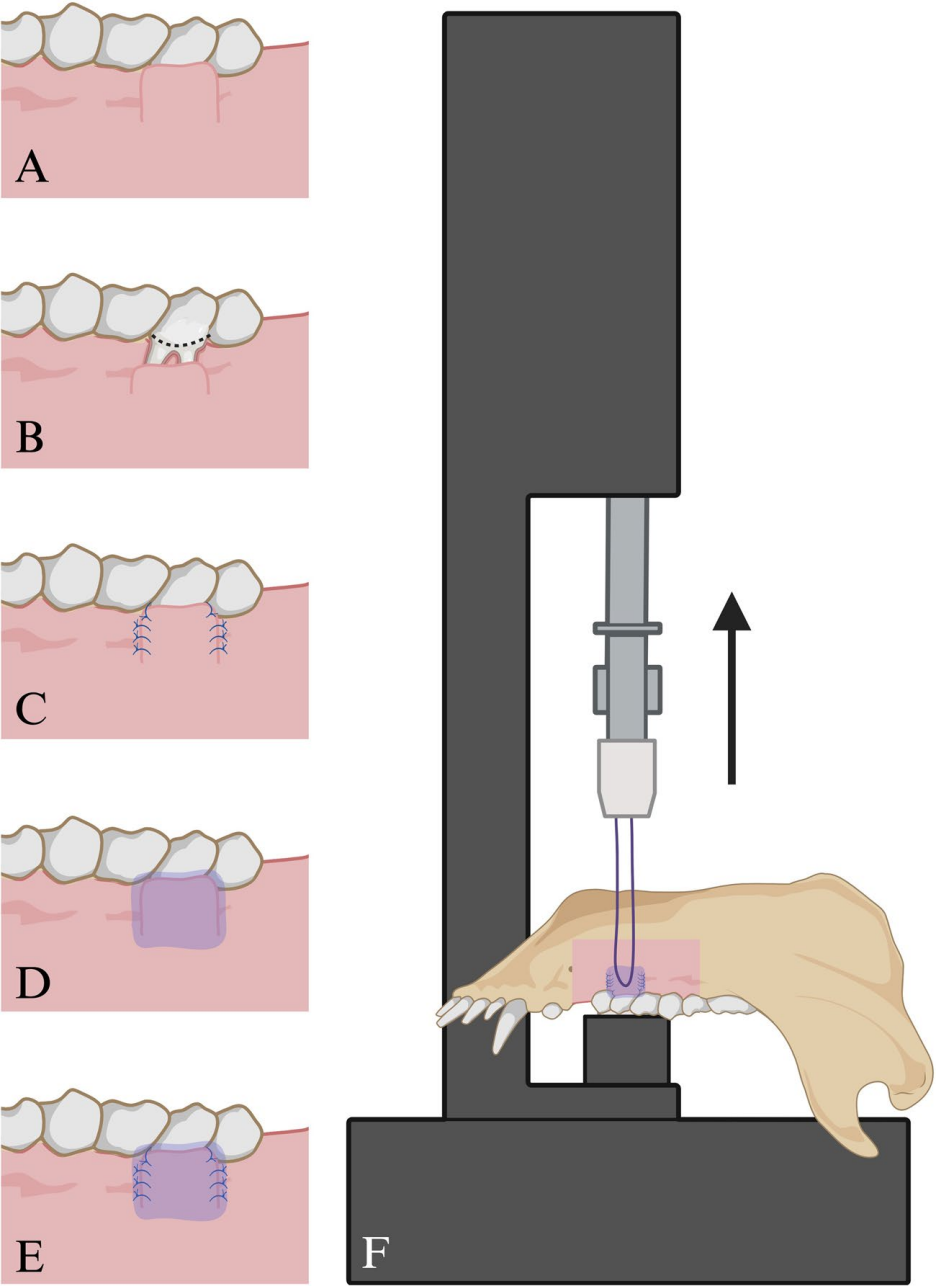
Overview of the study setup. **A** Incision of CAF for covering localized recession-type defects. **B** Marking the CEJ (black dashed line). **C** CAF fixed with sling and tag suture. Vertical releasing incisions were closed with single interrupted sutures. **D** CAF fixed with three layers of tissue adhesive. **E** CAF fixed with sling and tag suture and three layers of tissue adhesive. **F** Mandible fixed in the material testing machine with resorbable suture material anchored in the CAF serving as a suspension for the tensile measurement

Testing biomechanical characteristics of oral tissues and CAF was described in previous ex-vivo, cadaver, and in vivo studies [6, 19, 38]. At the third premolar (P3) of the mandible, a 3-mm gingival recession was created by performing a gingivectomy with a sharp 15c scalpel blade. The recession depth was controlled by a periodontal probe (PCP UNC 15, Hu-Friedy, Chicago, IL, USA), and the CEJ was marked with a dark and thin water-proof permanent marker. The CAF for localized recession-type defects was prepared as described by de Sanctis and Zucchelli [7]. In brief, two horizontal beveled incisions were made mesial and distal to the recession defect, located at a distance from the anatomical papillae equal to the recession depth plus 1 mm. Two vertical beveled incisions were made at the end of the two horizontal incisions extending 3–4 mm into the alveolar mucosa. The trapezoidal-shaped flap was elevated in a split-full-split approach in the coronal-apical direction until the flap was able to reach tension-free a level 1 mm coronal to the marked CEJ. To standardize the experimental set-up, the height and width of the trapezoidal-shaped flap were controlled by a periodontal probe to ensure comparable flap sizes in all mandibles. The anatomical papillae were then de-epithelized, and the surgical papillae of the flap were fixed with one of the following techniques: In group (I), CAF was fixed with sutures alone (sling and tag suture) by using synthetic monofilament 6-0 sutures (Prolene 6.0; Ethicon, Summerville, USA). Thereby, a double sling suture was placed at a distance of 2 mm from the gingival margins, followed by two simple interrupted sutures at the center of the papillae. In addition, three simple interrupted sutures directed apico-coronal were used for closing the vertical releasing incisions (Prolene 6.0) [6]. Distances between the sutures were controlled by a periodontal probe to ensure a comparable initial situation in all flaps. In group (II), CAF was fixed by tissue adhesive (three layers) over the CAF and the surrounding tissue. Fixation of the soft-tissue with CTA was performed by using anatomical forceps when the CAF was tension-free positioned at a level 1 mm coronal to the marked CEJ. In group (III), a combination of both procedures (I and II) was performed. Following flap fixation, it was ensured in all groups that the flap was positioned 1 mm coronal to the CEJ. Subsequently, braided resorbable suture material (Vicryl 3.0, Ethicon) was used for a single horizontal suture anchored in the central portion of the flap (including the periosteum) with a distance of 3 mm from the free gingival margin. This suture served as a suspension for the tensile measurement device (Fig. 1). Figure 2 gives an overview of the study setup. The following parameters were measured before and during the surgical procedure: recession depth on P3 at baseline (Rec 0 [mm]), recession depth on P3 after creating a 3 mm recession, measured at the mid-buccal site from CEJ to the gingival margin (Rec 1 [mm]), keratinized tissue width on P3 measured as the distance from the gingival margin to the mucogingival junction at the mid-buccal site (KTW [mm]), gingival thickness on P3 measured 2 mm apical to the gingival margin using a caliper (GT [mm]), distance from the occlusal margin of P3 and the CEJ at the mid-buccal site (OM-CEJ [mm]), distance from the occlusal margin of P3 and the position of the flap following suturing (OM-MG [mm]), the comparison of OM-CEJ and OM-MG allowed to control that the postsurgical position of the flap was 1 mm above the marked CEJ.

### Biomechanical testing

Marginal flap stability (MFS) against pull-off forces in the different groups (I–III) was tested using a universal material testing machine (Model 5942; Instron Pfungstadt, Germany) and the software BlueHill (version 2.25; Instron). Mandibles were clamped in a tensile grip jaw with the occlusal surface turned down. Then, the test sutures were tied to a loop and fixed to the testing machine. The pulling direction was upwards parallel to the long axis of the tooth. The uniaxial force was applied to the CAF at a constant strain rate of 0.5 mm/s under displacement control until the marked CEJ became visible. Maximum tensile force (N [Newton]) was measured at this point [39].

### Statistics

Statistical analysis was performed at the Institute for Medical Biostatistics, Epidemiology and Informatics (IMBEI) of the University Medical Center Mainz, Germany. Data was collected in MS Excel (Microsoft, Redmond, USA). Statistical analyses were conducted using R. To test the null hypothesis that the distributions of two continuous covariates do not differ, a Mann-Whitney *U* test with continuity correction was applied. Additionally, linear regression models were used to assess whether there is any correlation between the variables KT/GT and maximum tensile force.

## Results

### Maximum tensile force

**Fig. 3:**
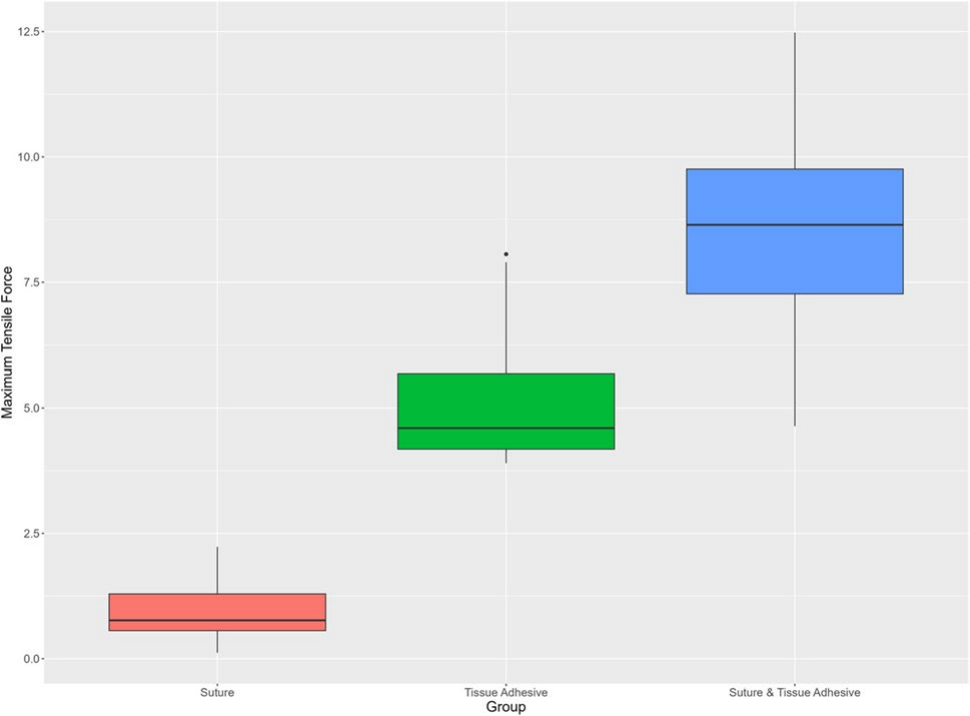
Maximum tensile force. *X*-axis: experimental groups, *Y*-axis: maximum tensile force [N]

**Fig. 4:**
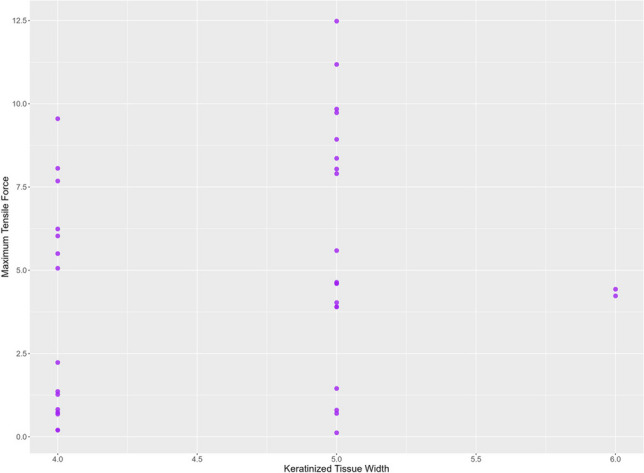
Correlation of maximum tensile force and keratinized tissue width

**Fig. 5:**
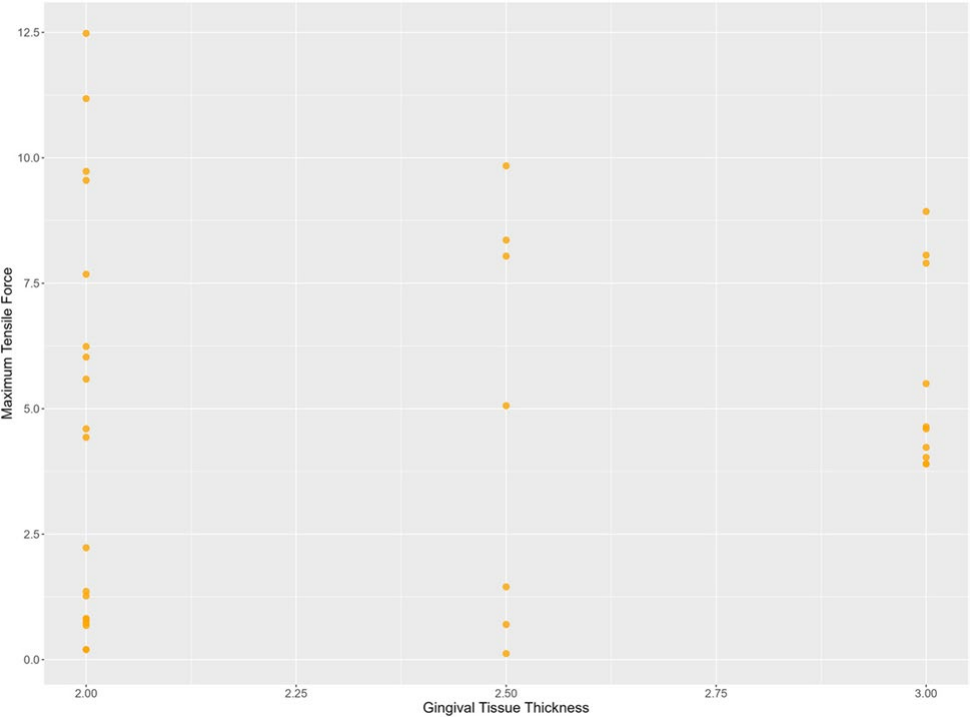
Correlation of maximum tensile force and gingival tissue thickness

The measurements of forces necessary to displace the gingival margin of the flap apically showed a tensile force of 0.88 ± 0.61 N for the suturing group, 5.20 ± 1.47 N for the tissue adhesive group, and 8.50 ± 2.30 N for the combined suture and tissue adhesive group. Analyzing differences between the experimental groups (I-III) demonstrated a significantly increased maximum tensile force for the tissue adhesive group (II) compared to the suture group (I) (*p*<0.001). A significantly increased maximum tensile force was found for the suture and tissue adhesive group (III) when compared to the suture group (I) (*p*<0.001). There was also a significantly increased maximum tensile force in the suture and tissue adhesive group (III) compared to the tissue adhesive group (II) (*p*<0.001) (Fig. 3). KT and GT were not significantly correlated with the maximum tensile force (*p*=0.19 and *p*=0.46, respectively) (Figs. 4, 5).

### Keratinized tissue width

**Fig. 6:**
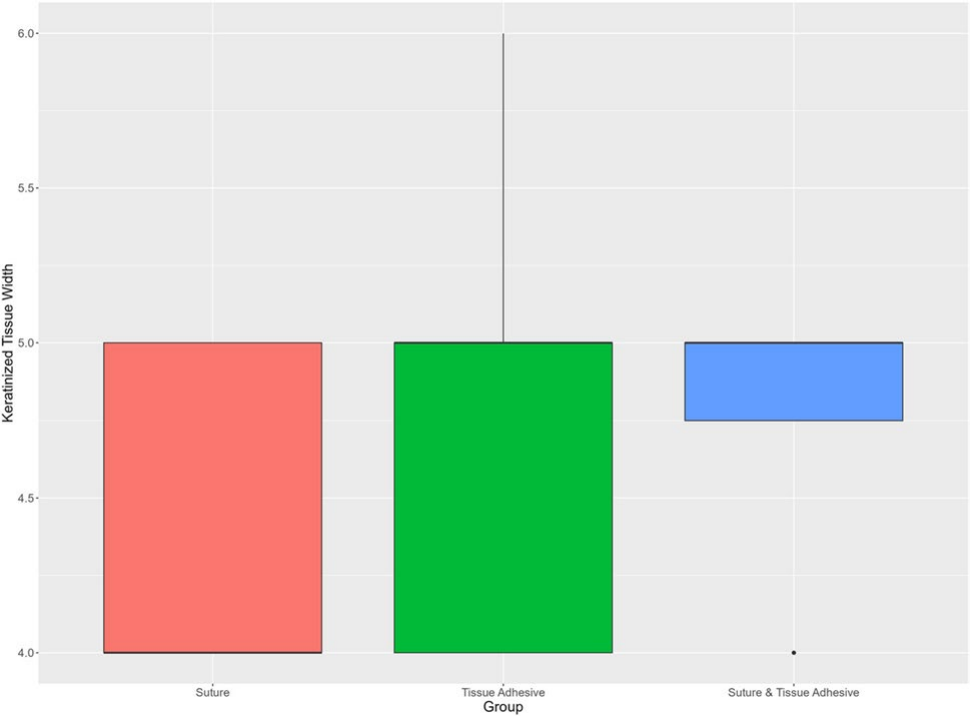
Keratinized tissue width. *X*-axis: experimental groups, *Y*-axis: keratinized tissue width [mm]

KTW was 4.33 ± 0.49 mm for the suture group, 4.83 ± 0.72 mm for the tissue adhesive group, and 4.75 ± 0.45 mm for the combined suture and tissue adhesive group. Comparing KTW between the respective experimental groups (I–III), the difference between the distributions of the suture group (I) and the tissue adhesive group (II) was not significant at the chosen significance level (*p*=0.076). There were also no significant differences between the tissue adhesive (II) and the suture and tissue adhesive group (III) (*p*=0.866). In contrast, a borderline significant difference (*p*=0.049) was observed between the suture group (I) and the suture and tissue adhesive group (III) (Fig. 6).

### Gingival tissue thickness

**Fig. 7:**
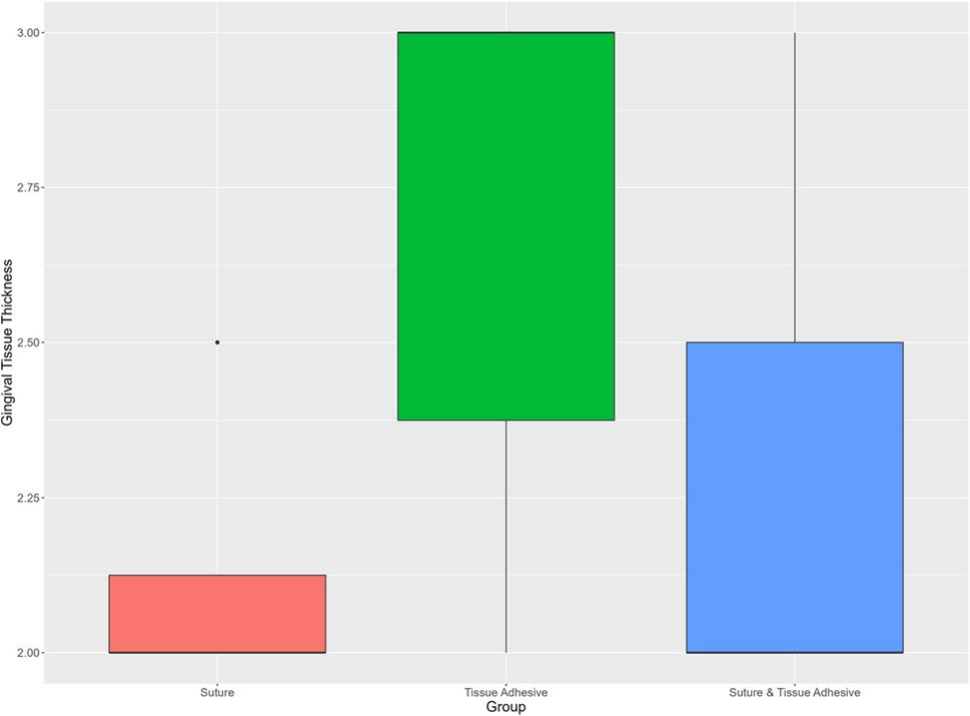
Gingival tissue thickness. *X*-axis: experimental groups, *Y*-axis: gingival tissue thickness [mm]

The GT was 2.13 ± 0.23 mm for the suture group, 2.71 ± 0.45 mm for the tissue adhesive group, and 2.29 ± 0.40 mm for the combined suture and tissue adhesive group. Comparing GT between the experimental groups (I-III), significant differences between the suture (I) and the tissue adhesive group (II) (*p*=0.003) were found. There were further significant differences between the tissue adhesive (II) and the suture and tissue adhesive group (III) (*p*=0.031). No significant differences were found between the suture (I) and the suture and tissue adhesive group (III) (*p*=0.313) (Fig. 7).

## Discussion

Since the introduction of the CAF technique, a variety of different root coverage procedures has been proposed. Nowadays, there is a large amount of evidence that the CAF + subepithelial connective tissue graft (SCTG) can be considered the gold standard for the treatment of recession-type defects [40, 41]. The outcome of this procedure is affected by several prognostic factors, such as patient-related factors, tooth/site-related factors, and technique-related factors [42]. Amongst all these factors, the soft tissue handling can be considered a key factor that is related to the skills of the operator. This includes the design of the flap, the tension of the flap, and the positioning of the flap [42]. Indeed, the postoperative position of the gingival margin in relation to the CEJ is considered an important factor in achieving complete root coverage following a CAF procedure [43, 44]. Moreover, a tight marginal flap adaptation is considered essential for promoting wound healing and blood clot stabilization [45, 46]. Most commonly, sutures are used for wound closure and stabilization of the wound margins since they provide adequate mechanical support to the healing of the tissues. As an alternative, cyanoacrylate-based tissue adhesives have been proposed for intraoral wound closure [27]. The results of the present study clearly demonstrate the ability of CTA to improve marginal flap stability following CAF when compared to suturing alone. This was found when CTA was used alone and when applied as an adjunct to suturing. A possible explanation for this finding is that after polymerization of CTA, a strong bonding and stabilizing effect is obtained over the entire extent of the flap compared to only punctual fixation with sutures. Indeed, cyanoacrylate tissue adhesives demonstrated to have strong bonding capabilities to biologic tissues when compared with other adhesives [47]. Moreover, the ability of CTA to adhere to the tooth surface might have contributed to an additional stabilization in the marginal tissue area in the present study. Thus, when referring only to MFS, the use of CTA alone seems to be sufficient for atraumatic CAF stabilization. This finding is further supported by previous research showing that CTA can be used as an alternative to sutures for stabilization following different flap procedures [48, 49]. Furthermore, several studies reported that the use of CTA compared to standard suture wound closure offers several advantages such as ease of use, faster application time, better hemostasis, aid in initial wound healing, less intraoperative and postoperative discomfort to the patients, antimicrobial properties, and decreased wound infections [33, 37, 50, 51]. Due to the hemostatic and anti-infective properties, the use of CTA seems also beneficial in patients with an increased risk of bleeding, diabetes, and immunodeficiency. From the clinicians’ point of view, the biggest advantage of CTA is that compared to suturing, traumatization of the tissue is avoided, thereby reducing the risk of tearing the flap and compromising vascularization. On the other hand, one must be aware that the present results obtained from a pig mandible model cannot be extrapolated to the clinical situation in humans in general. Thus, the soft tissue conditions used in the present pig jaw model may differ from the clinical situation in humans with respect to hydration, vascularization, tissue thickness, elasticity of the tissues, and muscle tension [6]. Moreover, the results of the present study are based on MFS measurements without a clinical setting evaluating the wound healing and the clinical outcomes. Thus, one might question if the high MFS obtained in the present study could be maintained over time in a clinical environment. It has been demonstrated that the strength of flap attachment to the root surface is weak during the first 2 weeks of healing [52, 53]. Therefore, it is necessary to ensure stability of the wound during this period until flap attachment to the root surface reaches a clinically adequate strength level. In this context, it has been shown that early suture removal (<10 days postoperatively) can negatively influence root-coverage outcomes following a CAF procedure [54]. It can be speculated if the sole use of CTA would be sufficient to ensure MFS over a 2-week period, and therefore, the combination with a suitable suturing technique seems reasonable. In the present study, the sling and tag (SAT) suture technique was used either alone or in combination with CTA. Tavelli et al. reported in a study on human cadaver heads with the SAT technique the highest MFS when compared with sling and simple interrupted sutures [6]. The obtained MFS of ~5 N with the SAT technique is much higher compared to the values in our study (0.88 N). This can be attributed primarily to the use of 5-0 sutures compared to 6-0 sutures in the present study. Further factors that may account for the difference include the used suturing material, bite size, tension of sutures, type and number of knots, cadaver model, and the type of tensile testing machine.

The analysis of data in the present study found no significant correlation between GT and initial MFS. Initial GT is considered a critical factor associated with complete root coverage with CAF procedures [55]. Thus, it was demonstrated that sites with an initial gingival thickness (≥ 1.2 ± 0.3 mm) demonstrated a higher chance of complete root coverage than those with GT < 1.2 mm. A thicker flap tissue may facilitate marginal adaptation in the CEJ area and provide more stability in the early healing phase. The uniform flap thickness (2–3 mm) in the present study might have precluded the finding of an interaction of GT and MFS. Similarly, no significant correlation between KT and MFS was found in the present study. The amount of KT apical to a recession defect is considered a critical factor for the selection of the root-coverage surgical procedure [56]. Moreover, a narrow band of KT was found to be associated with inferior treatment outcomes following surgical root coverage [57]. Indeed, the absence of apical KT makes flap handling, positioning, and fixation more difficult compared to clinical situations with the presence of at least 2 mm of KT. In the present study, a wide band of KT was present that might have contributed to flap manipulation and adaptation and therefore could be relevant for the overall treatment outcome in a clinical setting.

From an economic point of view, the cost-effectiveness of CTA compared to suture material must be discussed. Material costs (per unit) of the suture material (synthetic monofilament 6-0 sutures) and CTA used in this study are similar. In this context, further clinical studies must clarify whether on the one hand increased costs for using a combination of sutures and CTA are justified by improved clinical outcomes and on the other hand whether secondary costs, e.g., by postoperative complications, can be prevented by using a combination of sutures and CTA.

Apart from cyanoacrylate-based tissue adhesives, several other oral wound dressing materials have been proposed to accelerate healing, reduce postoperative pain, and prevent infection [58]. These include palatal stents, surgical sponges, platelet concentrates, zinc oxide–based dressings, collagen membranes, and hyaluronic acid [59, 60]. To what extent periodontal dressings are beneficial for intraoral wound healing and the impact of periodontal dressing composition has been the subject of debate [61]. Thus, it has been demonstrated that the use of different zinc oxide–based periodontal dressings was associated with increased plaque accumulation, irritation of the soft tissues, and more pain and swelling [61]. Saito et al. (2008) showed that non-eugenol zinc oxide–based periodontal dressings may induce an intense inflammatory response [62]. On the other hand, a most recent study demonstrated that the use of a surgical stent made from thermoplastic zinc-containing polymer granules provided significant benefits for wound healing parameters and patients’ postoperative morbidity in the early phase of palatal wound healing [63].

Since this is an ex-vivo study, some relevant clinical aspects cannot be taken into account, such as wound healing in an oral environment, muscle traction, long-term stability, and much more. The results and the CAF stability shown in this study can only be transferred to the clinical situation with limitations. To provide a more comprehensive evaluation, the results of this study should be re-evaluated in an in-vivo study and long-term clinical follow-up data should be generated to assess the persistence of benefits in clinical settings.

## Conclusions

Within the limitations of the present study, it can be concluded that CTA significantly improved MFS following a CAF procedure when compared to suturing. The addition of CTA to sutures further improved MFS compared to CTA alone and suturing alone.

## Footnotes

1 Online source: https://periacryl.com, last online access on September 2, 2023
